# Elevated oxytocin and noradrenaline indicate higher stress levels in allergic rhinitis patients: Implications for the skin prick diagnosis in a pilot study

**DOI:** 10.1371/journal.pone.0196879

**Published:** 2018-05-29

**Authors:** Jelena Gotovina, Christina L. Pranger, Annika N. Jensen, Stefanie Wagner, Oswald D. Kothgassner, Nadine Mothes-Luksch, Rupert Palme, Desirée Larenas-Linnemann, Jaswinder Singh, Ralph Mösges, Anna Felnhofer, Lisa-Maria Glenk, Erika Jensen-Jarolim

**Affiliations:** 1 Comparative Medicine, The Interuniversity Messerli Research Institute of the University of Veterinary Medicine Vienna, Medical University Vienna and University Vienna, Vienna, Austria; 2 Institute of Pathophysiology and Allergy Research, Center of Pathophysiology, Infectiology and Immunology, Medical University of Vienna, Vienna, Austria; 3 AllergyCare, Allergy Diagnosis and Study Center, Vienna, Austria; 4 Department of Child and Adolescent Psychiatry, Medical University of Vienna, Vienna, Austria; 5 Unit of Physiology, Pathophysiology and Experimental Endocrinology, University of Veterinary Medicine Vienna, Vienna, Austria; 6 Investigational Unit, Hospital Médica Sur, México City, Mexico; 7 Institute for Medical Statistics, Informatics and Epidemiology, Faculty of Medicine, University of Cologne, Cologne, Germany; 8 Department of Pediatrics and Adolescent Medicine, Medical University of Vienna, Vienna, Austria; Ospedale S. Corona, ITALY

## Abstract

**Background & Aims:**

The effects of acute stress on allergic symptoms are little understood. The intention of this clinical study was to study the effects of acute stress and related mediators in allergic rhinitis (AR), taking the wheal and flare reaction in skin prick testing (SPT) as a readout.

**Methods:**

19 healthy and 21 AR patients were first subjected to SPTs with grass pollen-, birch pollen- and house dust mite allergen extracts, histamine and negative control. Subsequently, participants were exposed to a standardized Trier Social Stress Test (TSST), followed by SPT on the contralateral forearm. Stress responders were identified based on the salivary cortisol levels and State-subscale of State-Trait-Anxiety Inventory (STAI-S). Blood samples were collected before and after TSST and adrenaline, noradrenaline, serotonin, oxytocin, platelet activating factor and prostaglandin D2 were analyzed by enzyme immunoassay (EIA).

**Results:**

SPT results of 14/21 allergics and 11/19 healthy who responded with stress after TSST were evaluated. No significant differences regarding SPT to allergens or histamine before and after the stress test could be calculated at the group level. But, the wheal and flare sizes after TSST increased or decreased substantially in several individuals, and unmasked sensitization in one “healthy” person, which could not be correlated with any mediator tested. The most significant finding, however, was that, independent of TSST, the baseline levels of oxytocin and noradrenaline were significantly higher in allergics.

**Conclusion:**

High baseline levels of noradrenaline points toward higher stress levels in allergic patients, which might be counterregulated by elevated oxytocin. Moreover, our data indicate that acute stress may have a significant influence on SPT fidelity in susceptible individuals.

## Introduction

Stress is a potent modulator of health [1] involved in exacerbation of asthma [2, 3] and atopic dermatitis (AD) [4, 5]. Atopic patients show defect stress coping mechanisms resulting in increased anxiety [6]. This may be due to an imbalance in the stress response involving the hypothalamic-pituitary-adrenocortical (HPA) axis with cortisol as main stress hormone, and the sympathetic adrenomedullary (SAM) responsiveness with adrenaline and noradrenaline. HPA axis function might be attenuated in patients with AD [7] and asthma [8], while SAM dysfunction causes increased catecholamine levels in AD patients [7].

In acute stress, there is a fine-tuned cross talk between the neural, endocrine and immune systems, resulting in a release of hormones and neuropeptides into the circulation to regulate both immune-mediated and neurogenic inflammation [2]. The major target of neuropeptides in the periphery are mast cells [9], which could thereby in a stress-related manner exacerbate inflammation [10]. AD highlights the skin as a neuro-immuno-endocrine organ, being both target and source of neuroendocrine mediators [11]. Upon IgE crosslinking, mast cells secrete pro-inflammatory and vasoactive mediators, prepacked as granules containing histamine and serotonin, or newly synthesized arachidonic acid metabolites including platelet activated factor (PAF). PAF together with histamine can increase the capillary permeability and induce wheal and flare reactions in the skin [12]. Histamine and serotonin contribute the prurigenic effect [13].

The neuropeptide oxytocin emerges as an inhibitor of the HPA axis and is shown to reduce anxiety [14, 15]. Besides its role in lactation and parturition, intra-nasally applied oxytocin combined with social support can reduce salivary cortisol responses to psychological stress [16].

More than 150 million Europeans suffer from allergies [17], there is therefore a constant need for diagnostic improvement. Although debated [18] skin prick test (SPT) still represents the most abundantly used test that objectively confirms sensitization in IgE-mediated allergic diseases [19]. It is a specific [20], sensitive [21] and inexpensive method which visualizes sensitization in the patient, important for his/her guidance. SPT as a cornerstone of allergy diagnosis is regulated by guidelines to ensure its diagnostic reliability and reproducibility [22]. Remaining diagnostic challenges include the variability between extracts from different manufacturers, beside the inter-subject variability [22]. Stress may induce exacerbation of allergic symptoms [23], or in other patients suppress symptoms via cortisol release. We thus hypothesized that acute stress might affect the actual skin hypersensitivity and the reliability of SPT.

The goal of our study was therefore to examine the effect of acute psychosocial stress on the IgE-mediated wheal and flare reactions elicited by pricked specific allergens or by histamine positive control, as a reductionist readout of sensitization. Our allergic and healthy cohorts were exposed to a standardized acute laboratory stressor, the Trier Social Stress Test (TSST) and stress responses were objectified by salivary cortisol determination. The individual perception of psychological stress in terms of subjective anxiety was evaluated using the State-subscale of the State-Trait-Anxiety Inventory (STAI-S) and selected mediators were determined in the blood.

## Materials and methods

### Pre-study: Left versus right forearm SPT

Left—right forearm sensitivity data were collected during a previous study [24]. The clinical study protocol was approved by the institutional review boards of the study centers, Centro Médico Nacional Siglo XXI, Mexico City, Mexico and Universidad Federal do Paraná, Curitiba, Brasil. Briefly, 14 grass pollen allergic patients (4 females, 10 males; 18–57 years) were tested with commercial timothy grass pollen extracts used for allergen immunotherapy including (A) TIM Soluprick^®^SQ 30 HEP/ml (ALK-Abelló, Madrid, Spain), (B) TIM Staloral ^®^300 IR/ml (Stallergenes, Antony, France), (C) Grazax^®^ 75 000 SQ-T (ALK, Denmark), (D) Oralair^®^ 300 IR (Stallergenes, Antony, France), all dissolved in 50% glycerin to a total volume of 1 ml per tablet (under good manufacturing practice conditons), and (E) TIM 10 000 BAU/ml extract (Greer Laboratories Inc., tenfold diluted from the commercially available 100 000 BAU/ml extract) as a reference. Extracts were tested in serial dilutions (concentrated, 1:3, 1:10 and 1:30 v/v). Histamine hydrochloride at 1mg/ml (a 10-fold lower concentration than in the subsequent major study) from ALK and vehicle (50% glycerin) were tested as positive and negative control respectively, resulting in a total of 22 test pricks. SPT was carried out simultaneously on both arms during two clinic sessions scheduled 2–3 weeks apart, to have a duplicate test result for all extracts on each arm. Twenty minutes after each SPT the total wheal surface for each testing point was marked with a felt-tip black pen and using surgical tape Transpore® (3M, Science applied to life, St. Paul, MN, US) transferred into the case report form. Files were processed digitally in the Institute for Medical Statistics, Informatics and Epidemiology, Faculty of Medicine, University of Cologne, where the wheal surfaces were calculated electronically using Scion Image (National Institutes of Health, Bethesda, MD). Finally, mean wheal surfaces of both tests in all 14 patients were calculated for each extract and its dilutions per arm.

### Main study

The study was approved by the ethics committee of the Medical University Vienna (EK Nr 1030/2015) and was conducted in AllergyCare, Allergy Diagnosis and Study Center Vienna (www.allergycare.at). Study subjects were recruited via advertisement, using an electronic questionnaire (http://www.soscisurvey.de/allergiestudie). All participants provided written informed consent to participate in the study. 40 age and gender matched study participants (21 males and 19 females; 21–34 years) met inclusion criteria and were classified into i) allergic patients group (n = 21), with doctor-diagnosed allergies and typical seasonal or perennial symptoms, and ii) a healthy participants group (n = 19), without doctor-diagnosed allergies and without symptoms. Skin prick tests during the study led to a re-classification of one person into the allergic cohort. Exclusion criteria: previously diagnosed psychological disorders or immunological diseases other than seasonal allergy; smoking, pregnancy, and current medication including steroids and non-steroidal anti-inflammatory drugs, as well as antidepressants.

### Study design

Upon arrival, participants were introduced to the study procedure **(S1 Fig)** and encouraged to wait comfortably in a relaxed atmosphere. Saliva samples were collected at 5 different time points: 2 baseline saliva samples upon arrival (45 min, and 5 min before the TSST) and 3 post-stressor samples were collected (5 min, 25 min, and 40 min after TSST). Psychological questionnaires were filled in before and after the TSST to assess personality traits in all test persons. Blood sample collection, following SPT was done shortly before (after a 50 min waiting period) and immediately after the TSST. The study was conducted between April 17 and May 26 2015, shortly after the birch pollen- and prior to the grass pollen- season in this year.

### Trier Social Stress Test (TSST)

The TSST, a protocol to induce moderate psychobiological arousal [25], is commonly applied to study stress responses in laboratory settings. The procedure consists of i) an anticipation period (5–10 min) in which participants are asked to prepare a free speech in front of a “committee” (constituted by the study team), followed by ii) the test period itself (10 min) in which participants must first deliver a speech and then complete mental arithmetic task. During the whole test procedure, the members of the committee maintain neutral facial expression without providing any verbal or non-verbal feedback, except for short instructions. It is well-documented that the exposure to this social evaluative threat will effectively stimulate the hypothalamic-pituitary-adrenal (HPA) axis and subsequent ACTH and cortisol secretion [26]. Overall, people subjected to this type of stressor will exhibit the largest increases in cortisol levels across cortisol-based laboratory stress studies [27].

### Psychological data

#### Evaluation of stress test by State-Trait-Anxiety Inventory (STAI-S)

The State-subscale of the State-Trait-Anxiety Inventory (STAI-S) [28] was used to determine the situational anxiety level, prior to and shortly after TSST, for each participant. It consists of 20-item state anxiety measures (e.g. “I'm nervous”) which were rated on 4-point-Likert-scale (“very”—“not at all”).

### Skin prick test (SPT)

SPT was performed on the right forearm before, and on the left forearm after the stress test according to guidelines [22], using extracts from birch pollen, grass pollen and house dust mites, histamine solution (10 mg/ml) for positive control, and a negative control (0.9% NaCl-solution), kindly provided by Roxall GmbH, Hamburg, Germany. Oral antihistamines were discontinued three days prior, and oral sympathomimetic treatments at least 12 hours before SPT. One drop of each allergen was placed 2 cm apart on the forearm and then pricked with a disposable metal lancet (Roxall GmbH). The reaction was read after 20 min and photographed, wheal and flares outlined with a pen and transferred with adhesive tape to a white paper sheet. SPT outlines were scanned and maximal and minimal diameters (mm), and therefrom area sizes (mm^2^) calculated by ImageJ (https://imagej.nih.gov/ij/). Analyses was done blinded by a single person to reduce the inter-observer-error. In addition to visual analyses, the subjective itching sensations prior and after the TSST were inquired by the patients, on a scale between 1 (no itch) to 10 (irresistible itch).

### Salivary cortisol analysis by EIA

Saliva samples were obtained using commercial sampling devices (Salivette®, Sarstedt, Germany) without saliva-stimulating additives. Study participants collected their saliva by a cotton roll into their cheek pouch for 60–80 sec. The collected material was immediately stored at -20°C. Prior to analysis, samples were thawed on ice and centrifuged at RT at 3000g for 15 min. Clear saliva samples were diluted 1:10 dilution and analysed using a highly sensitive double-antibody biotin-linked enzyme immunoassay (sensitivity of 0.2 pg cortisol /well) [29]. Average intra- and inter-assay coefficients of variation were less than 10% and 15%. To account for the circadian rhythm of cortisol, TSST and saliva sample collections were performed between noon and 6:00 PM.

### Plasma mediators: ELISA

Blood samples were drawn from the right arm before, from the left arm after the stress test using heparinized Vacutainer® vials, and centrifuged 15 min at 1000g, 4°C, and supernatants immediately stored at -80°C. Measurements were done in duplicates using enzyme-linked immunoassay (ELISA) kits according to the manufacturers′ instructions; noradrenaline and adrenaline: Labor Diagnostika Nord, Nordhorn, Germany (detection limits 20 pg/ml noradrenaline, 5.2 pg/ml adrenaline); serotonin: DLD Diagnostika, Hamburg, Germany (5 ng/ml); oxytocin: Enzo, Lausen, Switzerland (15 pg/ml); human platelet activation factor (PAF): Abbexa, Cambridge, United Kingdom (2.5 pg/ml); human prostaglandin D2 (PGD2): Bioassay Technology, Shanghai, China (5.02 ng/ml).

Blood cells were isolated from heparinized blood samples using Ficoll-Paque Plus 1.078 g/ml density gradient (GE Healthcare Biosciences, Uppsala, Sweden). PBMCs were deposited in a Neubauer chamber and counted using Primo Vert Microscope (Carl Zeiss, Germany). PBMC subpopulations were stained using the following fluorescent labelled antibodies: CD3-APC (clone SK7; eBioscience Inc.), CD4-PE-CY7 (clone SK3; BD Biosciences), CD8-PE (clone SK1; BD Biosciences) and CD14-FITC (clone MOPC-21; BioLegend), for 30 min at 4°C; after washing, 10.000 single cell events were acquired in BD FACSCanto II with FACSDiva Software (Becton Dickinson).

### Statistical analyses

Statistical analysis was performed either using Graph Prism (GraphPad Software Inc, San Diego, CA) or SPSS program (IBM SPSS version 20; IBM Corp., New York). More specifically, repeated measures in left-right SPT comparison and State-subscale of the State-Trait-Anxiety Inventory (STAI-S; Spielberg CD, 1983) were performed in SPSS program. The main study data were calculated using two-tailed Wilcoxon test for paired groups, for differences between allergic and non-allergic patients before/after TSST Mann-Whitney U-test for unpaired groups. The two-tailed Spearman rank order test was used to determine the nonparametric correlation between the salivary cortisol levels and the size of the HDM wheal area post-TSST. The statistical methods in the main study were selected by consulting “Statistics′ ambulance” of the University of Veterinary Medicine, Vienna, Austria.

## Results

### Pre-experiment: Comparing SPT wheal areas between the left and right forearms

**Fig 1:**
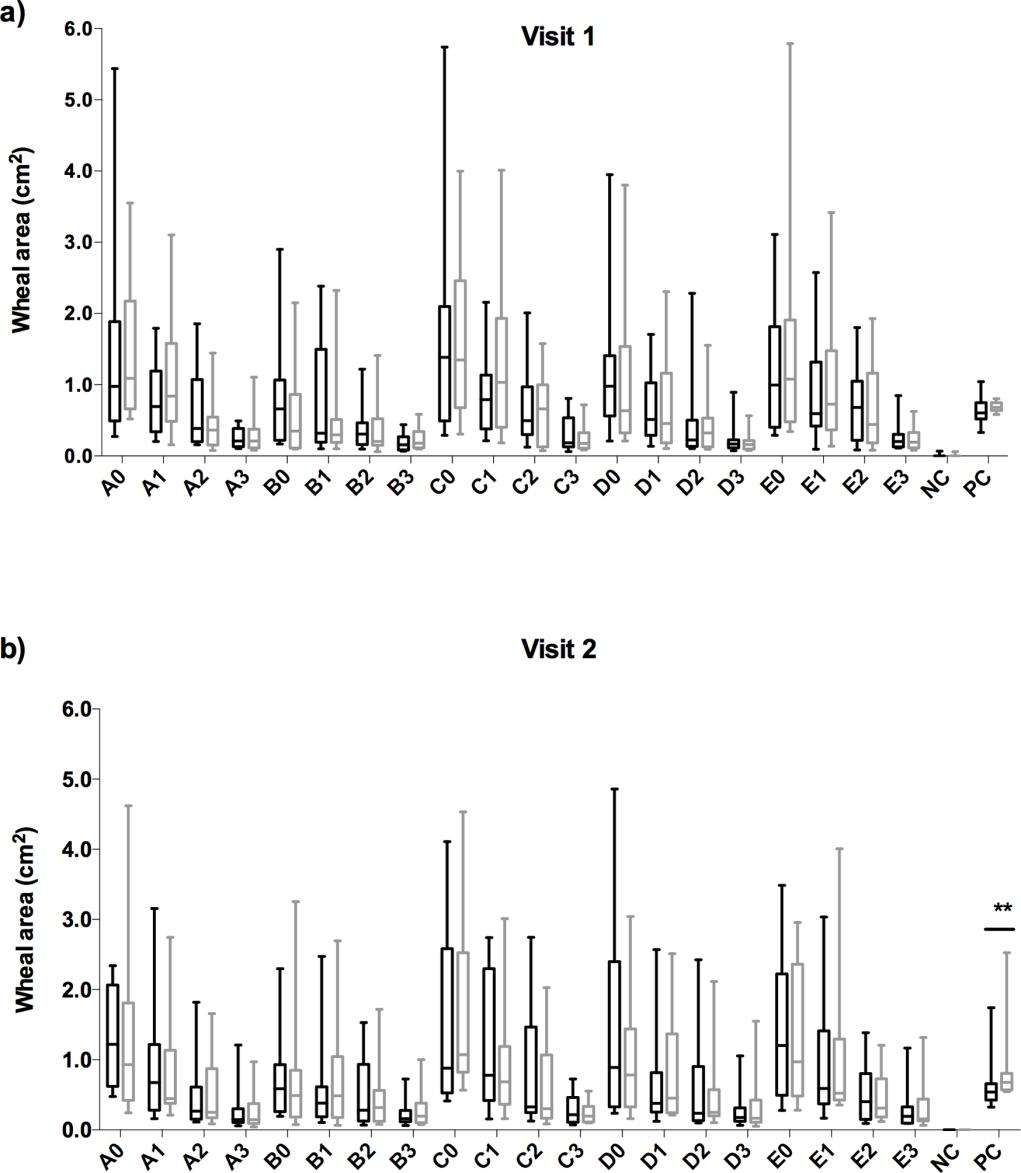
SPT left and right forearm comparison. Evaluation of the reliability of SPT with allergen extracts in 14 grass pollen allergic rhinitis patients at visit 1 **(a)** and visit 2 **(b)**. Box plots indicate median of SPT wheal surface areas (cm^2^) on the left (grey boxes) or right forearm (black boxes). 5 preparations of grass pollen extracts were used: A) Staloral® 300 IR/mL Phleum pratense pollen exact; B) Oralair® 300IR 5-grass pollen extract SLIT tablet, diluted in 1ml 50% glycerin; C) Soluprick® *Phleum pratense* pollen exact for SPT; D) Grazax® Phleum pratense pollen extract SLIT tablet, diluted in 1ml 50% glycerine; E) Reference Greer® 10.000 BAU/mL Phleum pratense pollen extract in different dilutions (E0: concentrated, E1: 1:3, E2: 1:10, E3: 1:30); vehicle negative control (NC) and histamine 1mg/ml positive control (PC). **p ≤ 0.01.

As we planned to perform SPT in each participant before and after TSST on the right, followed by the left hand side, we confirmed left-right comparability using data collected during a previous study [24]. In this study a total of 14 grass pollen allergic patients were simultaneously SPT tested on both forearms twice during 2 visits, being 2–3 weeks apart. Different dilutions of 5 timothy grass pollen exacts (A, B, C, D, E) commercially available for allergen immunotherapy were used and the wheal size recorded and evaluated by Scion Image **(Fig 1)**.

There was no significant difference in the wheal area sizes between left and right forearm, except for a trend with extract A) (**Fig 1,** A1) (Z = -1.915; p = 0.056) and B) (**Fig 1,** B3) (Z = -1.979; p = 0.048), only during the first visit **(Fig 1A)**. The positive histamine control showed significant difference in reactivity between left and right forearm, but only in the second visit (Z = -2.606; p = 0.009) **(Fig 1B)**. Based on the results of the left-right comparisons, SPT testing of study subjects on the 2 different forearms before and after the TSST was considered to render reliable results.

### TSST stimulated cortisol production in allergic and non-allergic subjects

After completion of the study protocol **(S1 Fig)**, salivary cortisol levels were assessed to control the effectiveness of the stress induction in our study. 14/21 of the allergic and 11/19 non-allergic participants responded in TSST with at least 10% increase in salivary cortisol and were thus classified as cortisol responders being eligible for further analyses **(Table 1)**.

**Table 1:** Characterization and clinical description of study participants.

Descriptors	Allergic	Allergic (CR) ^1)^	Healthy	Healthy (CR) ^1)^
**Number, %**	21 (52,50%)	14 ^2)^ (66,67%)	19 (47,50%)	11 (57,89%)
**Sex**	11 ♂ 10 ♀	7 ♂ 7 ♀	10 ♂ 9 ♀	5 ♂ 6 ♀
**Asthma**	4	3	—	—
**Total IgE > 100 UI/ml**	10	7	1	1
**Diagnosed allergies**	18	11	—	—
***Grass pollen***	8	6	—	—
***Birch pollen***	13	8	—	—
***HDM***	12	8	—	—
**Additional other allergens**	10	6	—	—
**AR symptoms ^3)^**	6	4	—	—
**Subjective perception of symptom severity ^4)^**	5	2	—	—

^1)^ CR (cortisol responders): individuals responding with at least 10% cortisol elevation after TSST.

^2)^ Three allergic patients underwent immunotherapy before study.

^3)^ Patients reporting to currently suffer from at least 3 typical rhinitis symptoms (sneezing, nasal pruritus, airflow obstruction or nasal discharge [30].

^4)^ Numbers of patients with a subjective perception of current AR symptoms > 3, on a scale ranging from 1 (minimal) to 5 (maximal symptom level).

**Fig 2:**
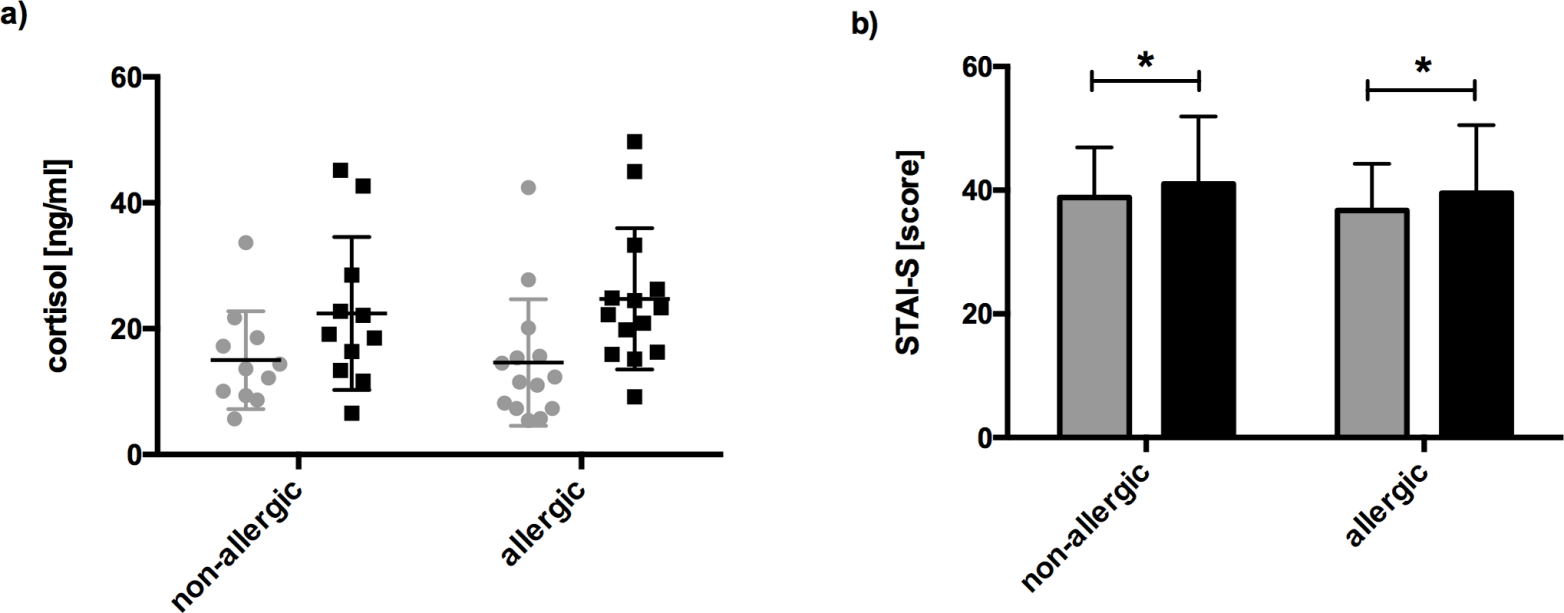
Verification of TSST-induced stress in study subjects. **a)** Salivary cortisol (means ± SD, ng/ml) in allergics (n = 14) and non-allergics (n = 11) who responded after the TSST; grey data points before, black after TSST; **b)** results of State-Trait-Anxiety Inventory (STAI-S) for self-reported anxiety in allergic (n = 20) and non-allergic participants (n = 19) before (grey boxes) and after the TSST (black boxes) (means ± SD); ***p ≤ 0.001. *P ≤ 0.05.

As illustrated in **Fig 2A**, the TSST induced a significant increase in salivary cortisol levels in both allergic (p≤ 0.001) and non-allergic individuals (p≤ 0.001). There was no significant difference in cortisol levels between allergic and non-allergic subjects before or after the TSST.

### STAI-S scale to determine anxiety in allergics after TSST

It has been previously proposed that allergics show higher level of anxiety compared to healthy individuals [6, 31]. To assess anxiety level in our test subjects we used the State-subscale of the State-Trait-Anxiety Inventory (STAI-S), a common self-report measure of anxiety [28]. Generally in all participants anxiety significantly increased after TSST (F(1,37) = 5.320; p = 0.027; par. eta^2^ = 0,126; ANOVA) **(Fig 2B)**. There was no statistically relevant difference between all allergic (n = 21) and non-allergic subjects (n = 19) prior and after TSST (F(1,37) = 0.445; p = 0.509; par. eta^2^ = 0,012). However, when only the cortisol responding subjects were analyzed, the mean score of the STAI-S scale differed between allergics (39.4 ± 7.39 before to 42.0 ± 10.95 after the TSST), and non-allergic (34.2 ± 8.28 before to 36.1 ± 10.00 after the TSST). Comparing these results to STAI normative data for working adults (35.7 ± 10.40) and patients with anxiety disorders (54.4 ± 13.02) [28, 32] revealed a higher mean anxiety (p = 0.01) in the cortisol-responding allergic cohort than in non-allergic cortisol responders.

Thus, all further analyses were performed only with the cortisol responding subjects (allergics: n = 14; non-allergics: n = 11). Altogether, significantly higher cortisol levels and increased state anxiety confirmed the successful stress induction by TSST in allergic and non-allergic subjects.

### Acute stress effects on SPTs with histamine and allergens

We next investigated the effect of the acute stress on SPTs by comparing wheal and flare surface areas before and after the stressor. The correlation of SPT results with previous doctor-diagnosed allergies and the mean wheal diameter to SPT allergen extracts used are summarized in **Table 2**.

**Table 2:** Comparison of SPT results in the allergic and healthy cohort before and after the TSST.

** **	**Allergic (n = 14)**^1)^	**Healthy (n = 11)**^1)^
**positive histamine prick**	100%	100%
**BP^2)^ allergy^3)^**	42,86%	—
**BP wheal**	57,14%	—
**before TSST** mean diameter mm, ± SD [range]	8,08 ± 2,15 [6,11–12,78]	—
**after TSST** mean diameter (mm, ± SD) [range]	7,59 ± 1,12 [5,96–9,51]	—
**GP^2)^ allergy^3)^**	57,14%	—
**GP wheal**	57,14%	—
**before TSST** mean diameter (mm, ± SD) [range]	6,39 ± 2,33 [3,71–10,29]	—
**after TSST** mean diameter (mm, ± SD) [range]	6,25 ± 2,38 [4,04–9,82]	—
**HDM^2)^ allergy^3)^**	57,14%	—
**HDM wheal**	71,43%	9,09%
**before TSST** mean diameter (mm, ± SD) [range]	6,64±1,48 [4,15–8,30]	—
**after TSST** mean diameter (mm, ± SD) [range]	8,23 ± 1,77 [6,15–12,30]	5,14

^1)^ CR: cortisol responders—individuals responding with at least 10% relative elevation of cortisol levels after TSST

^2)^ BP: birch pollen; GP: grass pollen; HDM: house dust mite

^3)^ Previous doctor-diagnosed allergy

All subjects responded with a wheal and flare reaction to SPT with the histamine positive control **(Table 2)**, but its size prior and after TSST did not vary significantly **(S2 Fig)**. Unexpectedly, not only the allergy cohort showed positive SPTs with the allergens: SPT with HDM extract led to positive reactions in 9.09% (1/11) of the subjects originally classified as non-allergic. This could also not be associated with atopic syndrome, as their total IgE levels were <100 IE/ml. Therefore, we suspect that HDM enzymes might directly have caused false positive reaction in sensible skin [33]. Also in the allergic cohort, SPT reactivity to HDM was higher (71,43%) than predicted by previously doctor-diagnosed HDM allergy (57,14%) **(Table 2)**. Analogously, while only 42,86% were previously doctor-diagnosed for birch pollen allergy, 57,14% reacted with a wheal and flare reaction to this allergen. Only in grass pollen, the previous diagnosis and SPT result were comparable (57,14%). 10 allergic and 1 non-allergic subjects (in the absence of any eczematous lesions) showed beside allergen-specific skin prick test results also elevated IgE, together suggesting an atopic background according to the definitions by AAAAI and WAO/EAACI (https://www.aaaai.org/conditions-and-treatments/conditions-dictionary/atopy; http://www.eaaci.org/attachments/304_English.pdf) **(Table 1)**. Elevated IgE levels did not correlate with SPT changes due to the TSST (data not shown).

**Fig 3:**
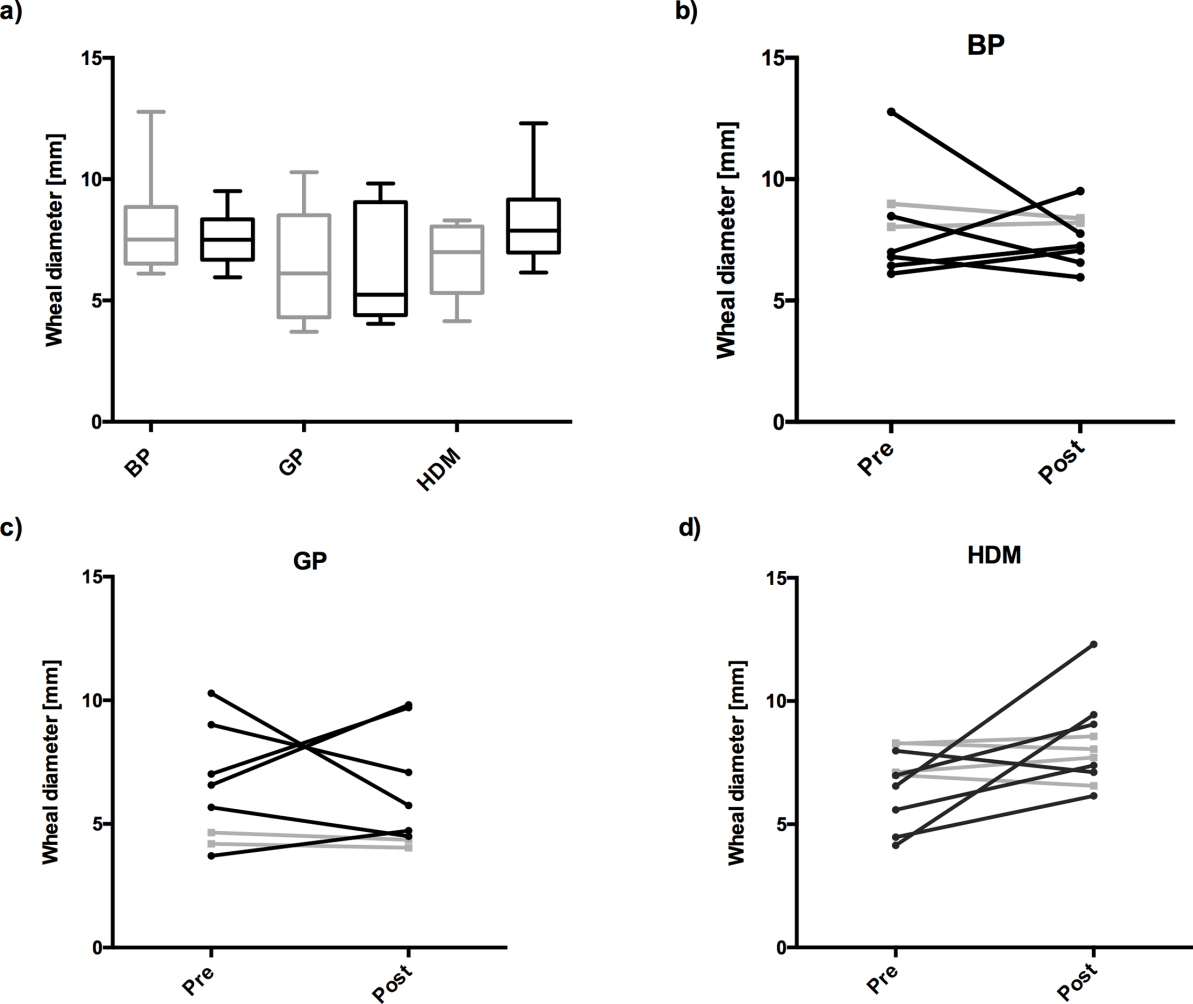
Changes in SPT wheal diameters before and after the TSST. **a)** Box plots illustrating changes in wheal diameters before (grey boxes) and after TSST (black) in the allergic cohort, in mm (y-axis); **b)** Individual changes in SPT wheal diameters to birch pollen (BP) (n = 8), **c)** grass pollen (GP) (n = 8), **d)** house dust mite (HDM) (n = 10), before (Pre) and after the TSST (Post); black lines in **b)-d)** indicate subjects responding with >10% relative change of reactivity, grey: less than 10% change.

The mean wheal diameters of SPTs with the three selected allergens (birch pollen, grass pollen and house dust mite) before and after the TSST in the allergic or non-allergic cohorts are shown in **Fig 3A** and the individual responses to birch pollen in **Fig 3B**, grass pollen in **Fig 3C** and HDM in **Fig 3D**.

**Fig 4:**
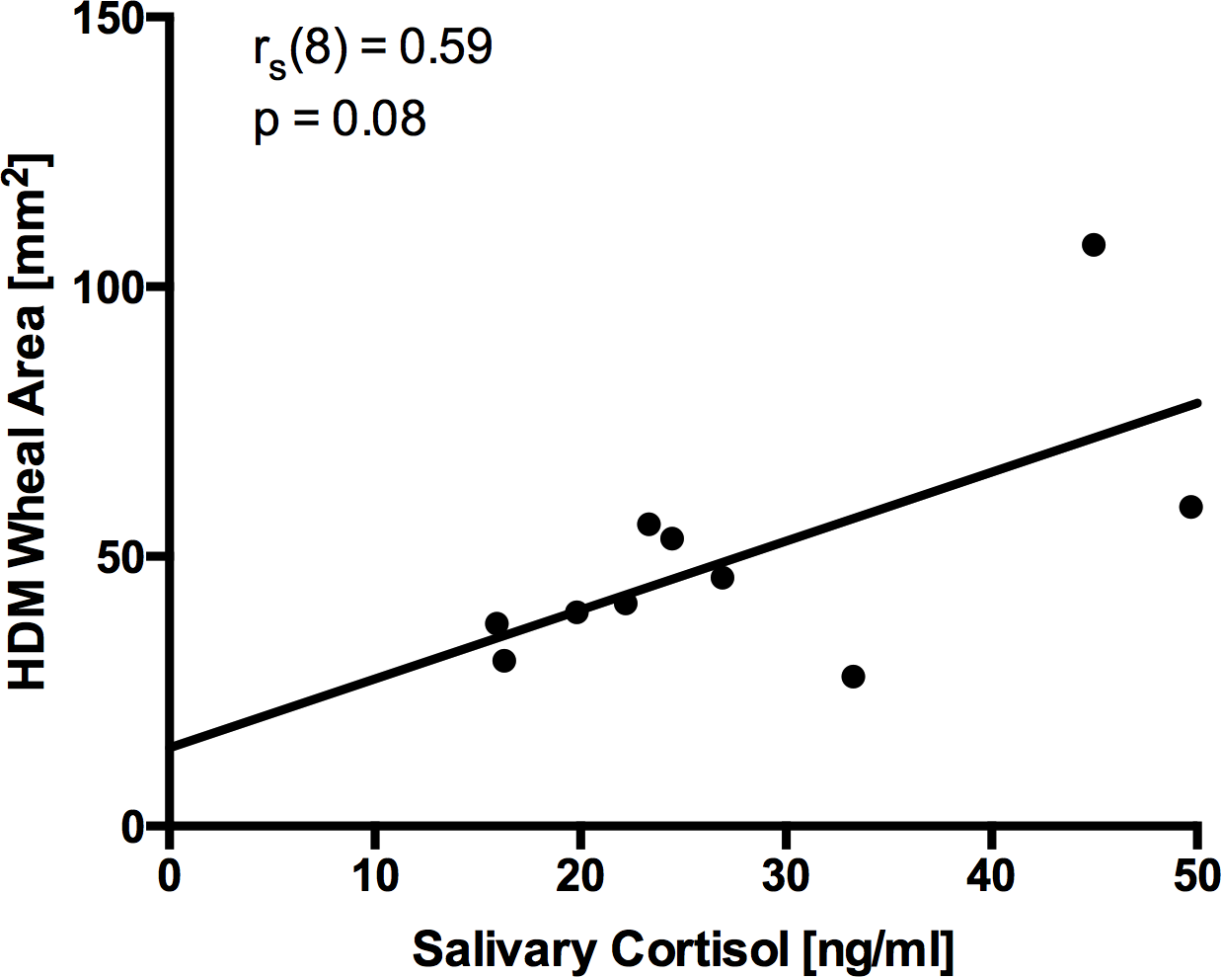
Spearman’s correlation analysis between HDM wheal area [mm^2^] and the salivary cortisol (ng/ml) Post-TSST. The solid line is a linear fit to the data with the Spearman correlation coefficient of r_s_ = 0.59.

No statistically significant difference before and after the stressor could be measured with any of the pricked allergens at the group level. While in some of the subjects acute stress showed no effect on SPT sensitivity, in other susceptible individuals the TSST did increase or decrease the wheal and flare response individually and depending on the allergen **(Fig 3B–3D)**. In some healthy subjects, the TSST unmasked sensitizations. This was, however, only the case with HDM SPTs **(Table 2)**. At the post TSST measurement there was a positive correlation between higher cortisol levels and larger HDM wheal area size with the tendency toward significance, r_s_ = 0.59, p = 0.08 **(Fig 4)**.

No significant differences were reported in the subjective itching sensations between the cohorts, and before or after the TSST (data not shown).

### Analyzing plasma mediators related to stress

**Fig 5:**
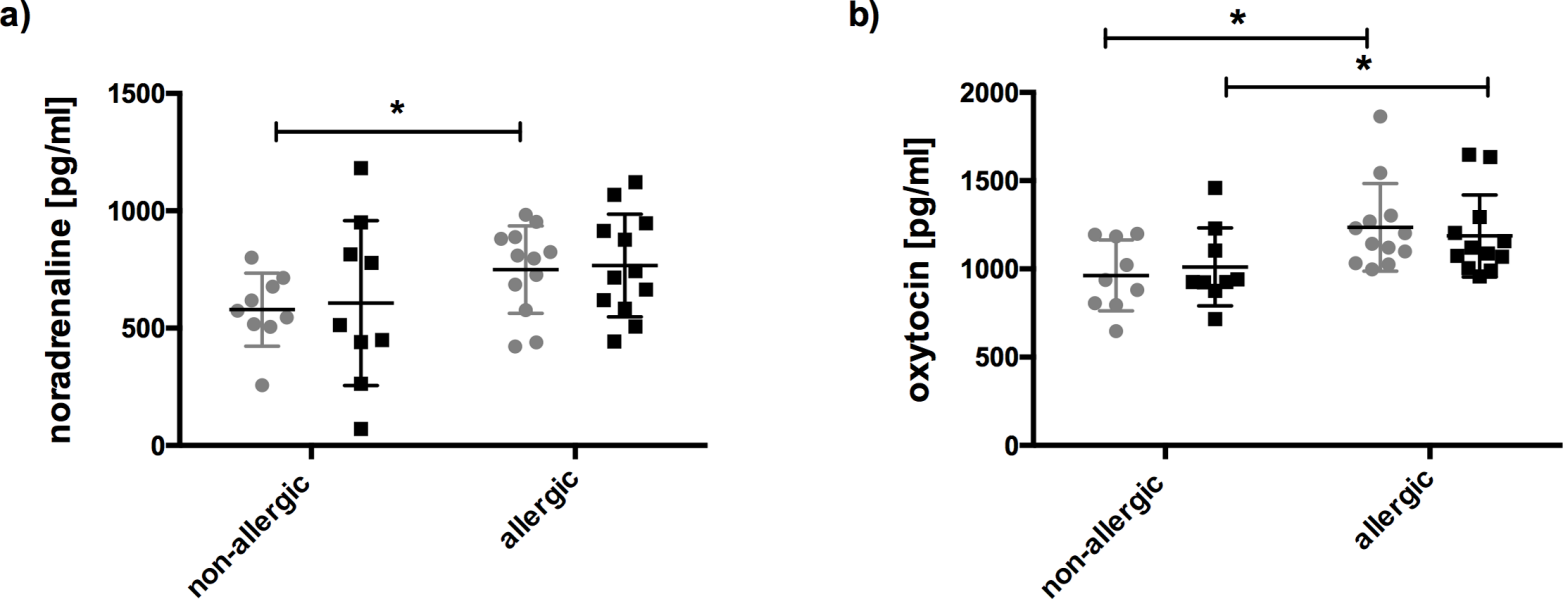
Significantly higher baseline levels of noradrenaline and oxytocin in allergic patients. Samples of cortisol-responding allergics (n = 12) and non-allergics (n = 9) were tested before TSST (grey dots) or after (black) for **a)** plasma noradrenaline and **b)** oxytocin levels. Data are represented as means ± SD; *p≤ 0.05.

To understand the individual differences in TSST-associated changes in SPT results, we next aimed to investigate mediators involved in central or peripheral stress responses. While none of the selected plasma mediators showed differences associated with the stress response **(S3 Fig)**, there were significantly higher baseline levels in plasma noradrenaline (p≤ 0.05) and oxytocin (F(1,19) = 5.628; p = 0.0284) in allergic compared to healthy subjects **(Fig 5)**; these were, however, not affected by the TSST.

## Discussion

Since most allergists stick to the current practice of skin testing before any serum tests for specific IgE are done, SPT remains the most widely used testing *in vivo* testing method for allergy diagnostics worldwide [19], and has been used to determine prevalence of allergic sensitization [34, 35], but not clinically relevant allergy [36]. We hypothesized that the accuracy of this real-time test might be dependent on variable factors not only from the operator-, but also from the patient-side. We aimed to investigate whether acute stress potentially could impact the accuracy of the readout of this, otherwise reliable *in vivo* test.

In the present paper the reliability of skin prick testing under stress exposure in human patients is addressed, as stress not only affects immune responses [37] but exacerbates asthma and AD [2–5]. We hypothesized that short-term acute stress could affect also the outcome of SPT, while IgE as a serum marker is constantly expressed [18].

Therefore, we planned in our study to compare SPTs in allergic and non-allergic subjects before and after a central laboratory stressor. In this procedure it was necessary to perform SPT within a short time interval twice in the same subject (**S1 Fig**), implying comparable skin reactivity on both left and right volar forearm. We took advantage of a data set generated during a previously published study [24] where 5 commercial grass pollen extracts (A-E) intended for allergen immunotherapy had been titrated on the skin simultaneously on both arms, and repeated in 2 visits (**Fig 1**). The data set collected in the pre-study convincingly demonstrated similar sensitivity between left and right forearm in the 14 tested grass pollen allergic patients, and was precondition for our main study.

Exposure to a standardized laboratory stressor, the TSST [25], resulted in an increase of salivary cortisol levels [27] in allergic and non-allergic subjects (**Fig 2A**), in 65,80% participants in total, in accordance with [38]. Observed individually different baseline levels of cortisol may be due to i) the circadian rhythm of cortisol secretion (saliva samples were collected between noon and 6:00 PM), ii) stress before or upon arrival at the study site, or iii) individual factors such as gender differences [39], personality factors [40, 41] or mood [42]. These factors however, did not affect the evaluation of changes in cortisol levels in association with the TSST.

There are conflicting reports that cortisol levels may be either blunted in AD [7] and asthma patients [8], or elevated in other allergic cohorts [32] but in our study the differences in cortisol levels between the allergic and non-allergic subjects did not reach significance. It was proposed that also the pollen season may interfere with the stress response in atopics due to a dysfunction of the HPA axis [43] although the majority of allergics in our study (72%) did not report symptoms during the test period which was between the birch and grass pollen season **(Table 1)**. There was also no difference in the total number of PBMCs, as well as in CD4^+^ and CD8^+^ T cells population and CD14^+^ cell population between allergic and non-allergic responders (data not shown).

The significant increases in cortisol levels were paralleled by significant induction of anxiety in both groups as determined by STAI-S [28], a widely applied psychological tool with a good internal consistency (Cronbach’s alpha 0.87–0.88). In agreement with others [6, 31] anxiety was highest in the allergic cohort, but lower than in patients with anxiety disorders [32].

Thus both cohorts showed similar stress responses upon TSST, correlating with anxiety scores and cortisol responses, the latter enabling a stratification of the cohorts into cortisol-responders only.

Extracts from most important allergen sources, birch pollen, grass pollen and house dust mite were used for SPTs in both cohorts. We hypothesized that acute stress could either enhance or diminish the wheal and flare reaction, as various stress and inflammatory mediators have the capacity to interfere with cutaneous mast cells. At the group level, no significant differences in the wheal and flare areas were observed after TSST between cohorts in histamine- **(S2 Fig)** and specific allergen- pricks **(Fig 3)**. However, in a number of individuals the wheal reactions were clearly enlarged or reduced after TSST **(Fig 3)**. After TSST particularly HDM extract was prone to not only increase skin reactions in HDM sensitized allergics **(Fig 3A)**, but also revealed reactions in one person out of 11 in the non-allergic cohort **(Table 2)**. Taken into consideration the tendency toward positive correlation between cortisol levels and HDM wheal area post-TSST **(Fig 4)**, the non-genomic rapid effect of increased cortisol levels might account for this effect. It is well accepted that at high concentrations, cortisol can be readily incorporated into plasma membrane altering its physicochemical properties [44]. This might interfere with the innate enzymatic function of HDM [33] which may be effective in atopic, susceptible skin [45]. Taken together, our study confirms the general reliability of SPTs in allergy diagnosis. However, our study also revealed that acute stress increased, unmasked, or reduced immediate type skin reactivity in a substantial number of SPT-tested individuals.

To understand the mechanism underlying the individually varying SPT variations after the TSST we analysed candidate mediators from the blood with a potential effect on peripheral mast cells or capillaries **(S3 Fig)**. The TSST protocol results in activation of both HPA and SAM axes and leads to an increase in plasma catecholamine levels [7, 25, 32], for which virtually all immune cells express adrenergic receptors, mainly of the ß2 subtype [46]. While in all study subjects TSST effectively induced cortisol and anxiety, we failed to determine stress-associated elevation of catecholamines. This may be due to their significant instability in plasma and methodological issues. Serotonin, PDG2 and PAF are mast cell- and platelet- derived mediators involved in flush [12], recruiting inflammatory cells [47], and together enhance capillary permeability by stimulating mast cells and smooth muscles [12]. Allergen exposure can cause an increase of PGD2 levels in the nasal mucosa of sensitized patients, in the skin of AD patients and in the airways of asthmatics [47]. Mast cells, monocytes and macrophages express oxytocin receptors [48, 49], which may interfere with local inflammatory responses, linking neuronal emotions and hypersensitivity. In the present study, we were not able to verify any of these mediators explaining individual differences in SPTs after TSST.

Notably, the allergic cohort had significantly higher noradrenaline and oxytocin baseline levels compared to non-allergic controls, which were not changed by stress **(Fig 5)**. Constantly elevated baseline levels of both adrenaline and noradrenaline were found in AD patients [7]. On the other hand, oxytocin, secreted from the posterior pituitary during stressful stimuli [50], is recently emerging as a regulator of stress responses in particular due its anxiolytic-like effects [51]. However, oxytocin is not elevated upon a stress test [52] and does not counterbalance cortisol responses during stress [52]. Studies in rats underline the importance of the crosstalk between noradrenergic and oxytocin-producing neurons known to be involved in a stress-mediated oxytocin release [50]. While central administration of noradrenaline stimulates oxytocin secretion into the circulation, application of oxytocin augments the release of noradrenaline, which can be blocked by oxytocin receptor antagonists [53]. We hence suggest that the elevated oxytocin levels in our allergic cohort at baseline may be regarded as a constant coping mechanism to control anxiety and elevated noradrenaline. To this end it is not clear whether this is related to the atopic predisposition, or whether it is a consequence of the strains associated with the allergic disease.

## Conclusions

This pilot study renders several important information. Overall, the acute stress has no statistically significant influence on SPT results, which confirms it as a valuable tool for the assessment of IgE-mediated hypersensitivities. However, stress and arousal may substantially affect the outcome of SPTs in susceptible individuals and should therefore be avoided. Among investigated mediators, the notion that baseline levels of oxytocin and noradrenaline were significantly elevated in allergics was novel, and might be interpreted as a constant elevated stress which allergic patients are experiencing and a simultaneous effort to counterbalance it by oxytocin production. The implications of this finding should be addressed in future studies.

## Supporting information

S1 Fig Scheme of the study. Participants arrived 15 min before the test onset (time point 0). Blood samples (B1, B2) and saliva samples (S1-S5) were taken, and skin prick tests (SPT1 and SPT2) was performed at indicated time points, before (pre) or after (post) Trier Social Test (TSST). The whole procedure was accompanied by psychological questionnaires on anxiety (STAI-S-1, and -2).
(TIF)

S2 Fig Changes in SPT wheal and flare diameters to histamine pricks upon the TSST. **a)** allergics (n = 14) and **b)** non-allergics (n = 11) were classified as stress responders based on their salivary cortisol levels. Individual wheal (left hand) or flare areas (right panels) are shown (y-axis in mm^2^), before (Pre) or after TSST (Post). Black lines indicate subjects responding with >10% relative change of reactivity, grey: less than 10% change.
(TIF)

S3 Fig Individual plasma levels of mediators before and after the TSST test. **a)** adrenaline, **b)** serotonin, **c)** PAF and **d)** prostaglandin D2. Grey: mean values of results before TSST, black: mean values after TSST; y -axis: levels of mediators/ml.
(TIF)
