# Supplementary figures and images for: Elevated oxytocin and noradrenaline indicate higher stress levels in allergic rhinitis patients: Implications for the skin prick diagnosis in a pilot study
Source: PLoS One. 2018 May 29;13(5):e0196879. doi: 10.1371/journal.pone.0196879 (PMC5973608; doi:10.1371/journal.pone.0196879)

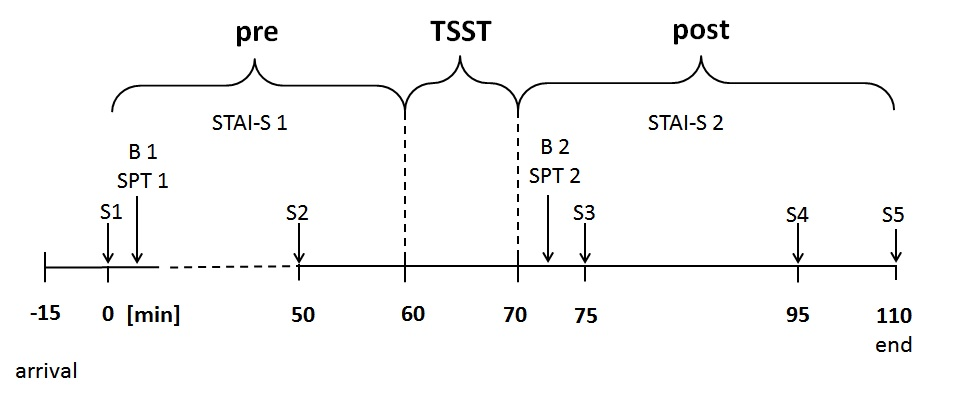

Supplement: S1 Fig — Participants arrived 15 min before the test onset (time point 0). Blood samples (B1, B2) and saliva samples (S1-S5) were taken, and skin prick tests (SPT1 and SPT2) was performed at indicated time points, before (pre) or after (post) Trier Social Test (TSST). The whole procedure was accompanied by psychological questionnaires on anxiety (STAI-S-1, and -2). (TIF) [file pone.0196879.s001.tif]

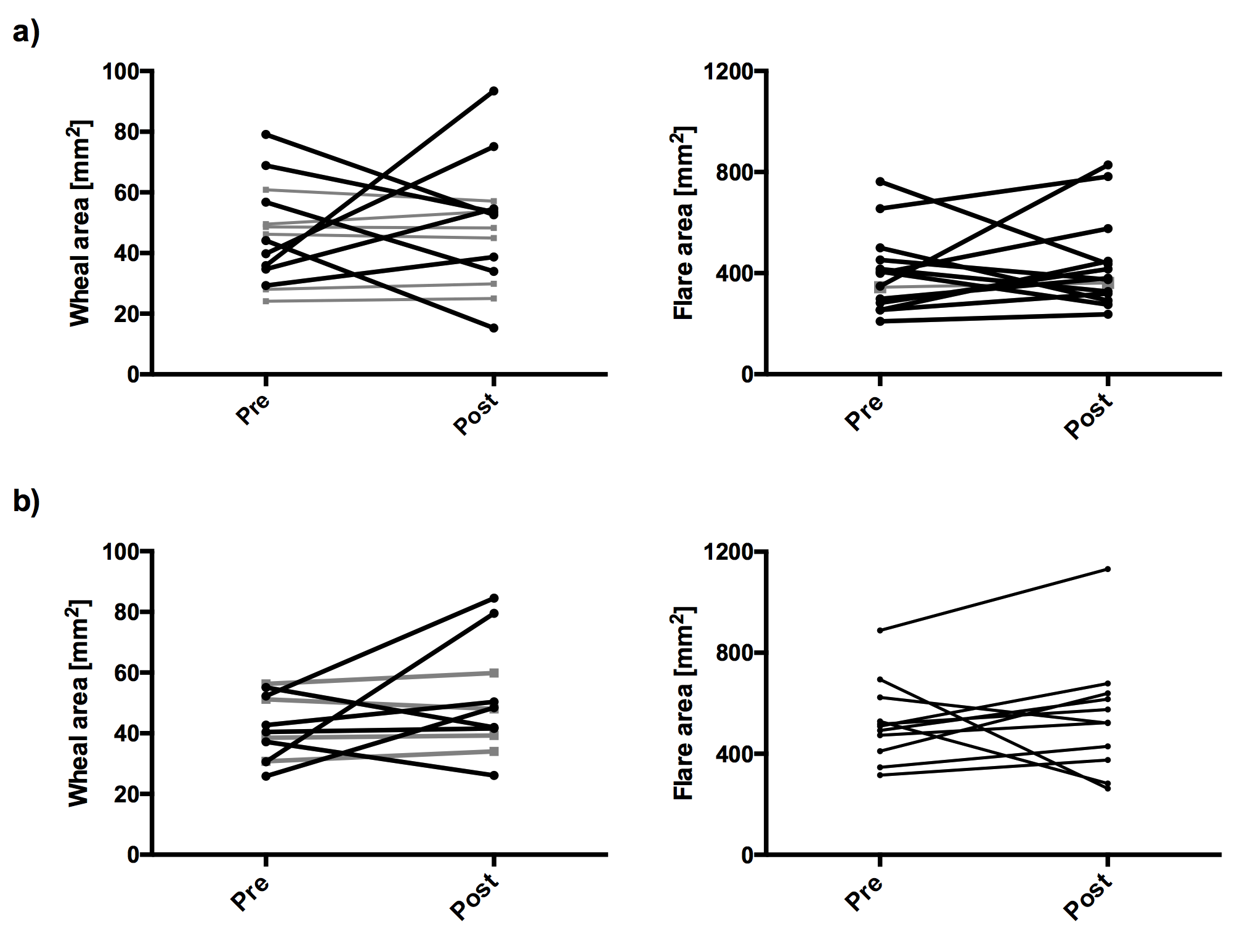

Supplement: S2 Fig — a) allergics (n = 14) and b) non-allergics (n = 11) were classified as stress responders based on their salivary cortisol levels. Individual wheal (left hand) or flare areas (right panels) are shown (y-axis in mm2), before (Pre) or after TSST (Post). Black lines indicate subjects responding with >10% relative change of reactivity, grey: less than 10% change. (TIF) [file pone.0196879.s002.tif]

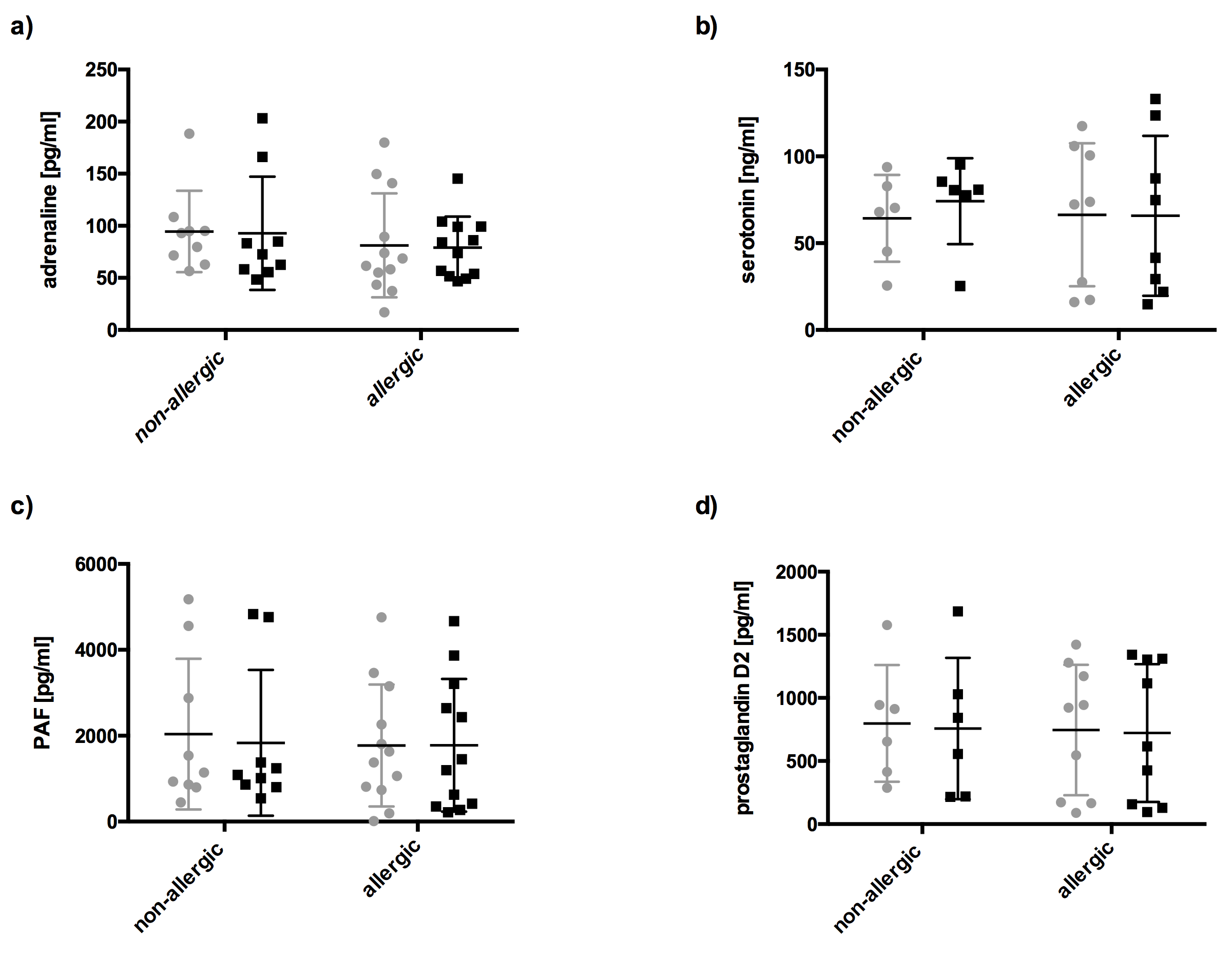

Supplement: S3 Fig — a) adrenaline, b) serotonin, c) PAF and d) prostaglandin D2. Grey: mean values of results before TSST, black: mean values after TSST; y -axis: levels of mediators/ml. (TIF) [file pone.0196879.s003.tif]
